# Maternal healthful dietary patterns during peripregnancy and long-term overweight risk in their offspring

**DOI:** 10.1007/s10654-020-00621-8

**Published:** 2020-03-17

**Authors:** Susanne Strohmaier, Leonie Helen Bogl, A. Heather Eliassen, Jennifer Massa, Alison E. Field, Jorge E. Chavarro, Ming Ding, Rulla M. Tamimi, Eva Schernhammer

**Affiliations:** 1 Channing Division of Network Medicine, Brigham and Women’s Hospital and Harvard Medical School, Boston, MA USA; 2 Leibniz Institute for Prevention Research and Epidemiology - BIPS, Bremen, Germany; 3 Department of Epidemiology, Center for Public Health, Medical University of Vienna, Kinderspitalgasse 15, 1. Stock, 1090 Vienna, Austria; 4 Department of Nutrition, Harvard T.H. Chan School of Public Health, Boston, MA USA; 5 Department of Epidemiology, Brown University, Providence, RI USA; 6 Department of Epidemiology, Harvard T.H. Chan School of Public Health, Boston, MA USA

**Keywords:** NHSII, GUTSII, Maternal diet, Pregnancy diet, Perinatal programming, DOHaD, Obesity

## Abstract

Adherence to healthful dietary patterns is associated with lower body mass index (BMI) in adults; however, whether maternal diet quality during peripregnancy is related to a lower overweight risk in the offspring remains to be elucidated. We investigated the associations between the Alternate Healthy Eating Index (AHEI), Alternate Mediterranean Diet (aMED) and Dietary Approach to Stop Hypertension (DASH) during peripregnancy and offspring weight outcomes in a study including 2729 mother–child pairs from the Nurses’ Health Study II and offspring cohort Growing Up Today Study II. Children, 12–14 years at baseline were 21–23 years at the last follow-up. Overweight or obesity was defined according to International Obesity Task Force (< 18 years) and World-Health-Organization guidelines (18 + years). Maternal dietary patterns were calculated from food frequency questionnaires. Log-binomial models were used to estimate relative risks (RR) and 95% confidence intervals. In models adjusted for sex, gestational age at delivery and maternal total energy intake, greater maternal adherence to aMED and DASH, but not AHEI, was associated with lower overweight risk in the offspring (RR_Q5 vs Q1_ = 0.82 [0.70–0.97] for aMED and 0.86 [0.72–1.04] for DASH, *P* for trend < 0.05 for both). After additional adjustment for maternal pre-pregnancy lifestyle factors and socio-demographic characteristic, none of the diet quality scores were significantly associated with offspring overweight risk. Maternal pre-pregnancy BMI did not modify any of these associations. In this population of generally well-nourished women, maternal healthful dietary patterns during the period surrounding pregnancy were not independently associated with offspring overweight risk at ages 12–23 years.

**Electronic supplementary material:**

The online version of this article (10.1007/s10654-020-00621-8) contains supplementary material, which is available to authorized users.

## Introduction

The United States has one of the highest childhood obesity prevalence in the world, with about 1 in 3 children and adolescents classified as having overweight and 1 in 6 as having obesity [1]. Due to the numerous negative health consequences of childhood obesity, such as increased morbidity and mortality in adult life, it represents one of the most serious public health challenges of the 21st century [2].

While it is known that genetics and the environment are related to the development of obesity [3], more research is needed to better understand the mechanisms involved in the development of obesity. With regard to environmental factors, suboptimal intrauterine conditions during sensitive periods of development have been suggested to permanently influence long-term disease risk in the offspring, including obesity and metabolic disease [4–7]. Perhaps the most influential human studies have been on the Dutch famine, clearly demonstrating that men and women conceived during the Dutch Hunger Winter in 1944–1945 who had been exposed to the famine during the first half of pregnancy had higher rates of obesity in adulthood [8, 9]. However, food shortages are currently not a persistent problem in industrialized countries, where an ever-increasing number of women enter pregnancy overweight or obesity [10, 11]. While research on maternal health behaviors during pregnancy and offspring health outcomes is still in its infancy, the findings so far suggest that a higher maternal pre-pregnancy body mass index (BMI) [12], excess gestational weight gain [12], gestational diabetes mellitus [13], and an unhealthy maternal lifestyle before pregnancy [14] increase the long-term risk of overweight and obesity in their offspring.

One additional maternal factor that might influence overweight and obesity risk in the offspring is maternal dietary intake during pregnancy. Animal models conducted under isocaloric conditions have shown that maternal high-protein [15] or high-fat intake [16] during critical periods of fetal development can “program” the offspring for a range of long-term metabolic consequences, including, for example, obesity, insulin resistance and decreased total energy expenditure.

Experimental studies suggest that moderate changes in maternal nutrition around the time of conception could signal life-long epigenetic changes in genes regulating energy balance in the hypothalamus [17], lending biological plausibility to the effects of early-life nutrition on offspring weight outcomes.

While certain individual nutrients and food groups during pregnancy have previously been associated with adiposity risk in the offspring [18–22], research has shifted towards dietary pattern approaches that take into account the potential interactions and synergies of nutrients within the habitual diet [23]. To measure the healthfulness of dietary patterns, three commonly used scores are the Alternate Healthy Eating Index 2010 (AHEI), the Alternate Mediterranean Diet (aMED), and the Dietary Approach to Stop Hypertension (DASH) [24]. Adherence to healthful dietary patterns, as assessed by these three diet quality scores, has been associated with less weight gain, especially in younger women and individuals with overweight [25, 26].

Some initial studies have also explored maternal healthful dietary patterns in relation to pregnancy outcomes and offspring health, describing associations with lower risk of e.g., gestational diabetes mellitus [27] or delivering a fetal growth-restricted infant [28]. However, previous studies did not find consistent associations between maternal healthful dietary patterns during pregnancy and childhood overweight risk in 4–10 year old children [29–31]. No study, to our knowledge, has examined maternal dietary patterns in relation to offspring weight beyond that age range. To address this research gap, we examined whether a higher adherence to healthful dietary patterns during and around pregnancy is related to a lower overweight risk in their offspring during childhood through early adulthood.

## Materials and methods

### Study population

For the present analysis, we utilized information on mother–child pairs; mothers were enrolled in the Nurses’ Health Study II (NHSII) and their offspring participated in the Growing Up Today Study II (GUTSII). NHSII was established in 1989, when 116,429 female nurses in the US, aged 25–42 responded to an initial questionnaire regarding their lifestyle and health. The study is still ongoing and follow-up questionnaires are mailed biennially to collect updated information on risk factors and major diseases. GUTSII started in 2004, when 17,280 children born between 1987 and 1995 to NHSII participants, received invitation letters and questionnaires, after obtaining maternal consent. A total of 10,918 children completed the original questionnaire and were sent follow-up questionnaires on health, lifestyle and growth indicators in 2006, 2008, 2011 and 2013. The current study comprises all mother–child pairs where the mothers completed a dietary assessment in 1991 as part of the NHSII study and the FFQ covered at least part of the pregnancy with their child who eventually joint GUTSII; thus reducing the sample to 2729 mother–child pairs (see exclusion details below). The study has approval from the Committees on the Use of Human Subjects in Research at the Brigham and Women’s Hospital and the Harvard T.H. Chan School of Public Health (Boston, MA, USA). Returning the baseline self-administered questionnaire was assumed to imply informed consent in both cohorts.

### Ascertainment of dietary patterns among mothers

Information on maternal dietary intake was collected for the first time in 1991 (and every 4 years thereafter) using a validated 131-item semi-quantitative food frequency questionnaire (FFQ) asking about a mother’s usual diet over the past 12 months. Briefly, a commonly used unit or portion size (e.g. piece, or oz.) was specified for each food and women were asked to report how often, on average, they consumed each type of food or beverage over the past year, with response options ranging from ‘never or less than once per month’ to ‘6 or more times per day’. Nutrient intakes were calculated by multiplying the frequency of intake for each food item by its nutrient content and summing nutrient contributions across all food items. A nutrient database derived from the US Department of Agriculture served as a source for the nutrient content of each food item [32]. Validation studies comparing intakes assessed by questionnaire with multiple diet records showed that this FFQ provides reasonable valid estimates for intakes of a wide range of dietary variables, with mean correlations coefficients of around 0.5 for different foods and nutrients [33–35]. Based on the FFQ information we could calculate scores that capture adherence to the AHEI, the aMED and DASH dietary patterns. For all 3 dietary patterns, higher scores represent higher adherence to the particular dietary pattern.

The AHEI was based on a comprehensive literature review and includes foods and nutrients that have consistently been associated with lower chronic disease risk [36]. In total, the intakes of 11 components are included and each component is scored from 0 (worst) to 10 (best). For 6 components (vegetables excluding potatoes, fruit, whole grains, nuts and legumes, long-chain omega-3 fats, polyunsaturated fat) higher intakes obtain higher scores while for 4 components (sugar-sweetened beverages and fruit juice, red/processed meat, trans fat and sodium) lower intakes obtain higher scores. For alcohol, moderate intakes were scored highest. All the component scores are summed to obtain a total AHEI score, which ranges from 0 to 110.

The aMED score was adapted for the US population from a previously published pattern by Trichopoulou et al. [37], based on the traditional Mediterranean diet. It was derived based on the following 9 components: vegetables (excluding potatoes), legumes, fruit, nuts, whole grains, red and processed meat, fish, alcohol, and the monounsaturated-to-saturated fat ratio. Participants were given 1 score point for being below the median intake for red and processed meat. For alcohol, one point was assigned if the intake was within the range of 5–15 g/d. For the remaining items, one point was given if the intake was above the median intake. Otherwise, they received 0 points. The components scores are summed and total aMED scores range from 0 to 9 [38].

The DASH score was developed based on foods that were either emphasized or discouraged in the DASH trial, originally put forth by Sacks et al. [39] for the reduction of blood pressure. The points for each of the 8 components were based on the participant’s quintile ranking. For fruits, vegetables, nuts and legumes, low-fat dairy and whole grains, participants in the lowest quintile were awarded one point and those in the highest quintile were awarded 5 points. For sodium, red and processed meat, and sugar-sweetened beverages the scoring was reversed. Again, the individual component scores are summed to give an overall DASH score with a possible range from 8 to 40.

### Ascertainment of weight outcomes among offspring

Offspring birth weight was collected on the GUTSII mothers’ questionnaire in 2009. The validity of maternal recall of offspring birth weight recall was found to be excellent [40]. In the 2004 GUTSII baseline questionnaire, participants, then aged 10–17, were asked to recall their body size at age 5. They were presented with eight pictograms (‘somatotypes’) and asked to select the one that would most accurately represent their body shape within a range from 1 = most lean to 8 = most obese. For the purpose of our analyses, we created a binary outcome, defining children with a reported body shape larger than the median of the distribution as cases. Further, self-reports on offspring weight and height were collected on each GUTSII follow-up questionnaire. Definitions for normal weight, overweight and obesity throughout follow-up were based on the age-and sex specific cutoffs from the International Obesity Task Force for participants aged 18 years or younger [41]. Beyond age 18, definitions were based on the standard World Health Organization cutoffs (i.e., BMI between 25 and 29.9 kg/m^2^ overweight and BMI > 30 kg/m^2^ obesity). We considered the outcome ‘ever having overweight or obesity’ and the more severe outcome ‘ever having obesity’ by defining participants as cases if they ever fell into the described categories at any given time during follow-up [14].

### Ascertainment of covariates

Total energy intake was derived from the FFQ, described above. Maternal age at birth of the included child was calculated based on mother’s and child´s birth dates. Smoking status and physical activity before pregnancy were based on the information given on the 1989 NHSII questionnaire. Likewise, pre-pregnancy BMI was based on height and weight measures reported in 1989 for women who were not pregnant at the time of the questionnaire return. Husband’s education, asked in 1999, was considered a proxy for socioeconomic status. Information related to the individual pregnancies was obtained from the lifetime pregnancy assessment in 2009, particularly the number of previous pregnancies, pregnancy-related hypertension, pre-eclampsia, gestational diabetes, mode of delivery, gestational age at delivery and pregnancy multiplicity. If mothers did not return the 2009 questionnaire, a missing indicator was introduced. Children´s sex and age were obtained from the baseline GUTSII questionnaire in 2004. For detailed covariate categories used in analyses, we refer to Table 1 and the footnotes of Tables 2 and 3, and all supplemental Tables.

**Table 1:** Maternal and offspring characteristics by quintiles of dietary patterns during peripregnancy, for 2729 mothers and children, born between 1990 and 1992, enrolled in the Growing Up Today Study 2

	AHEI-2010			aMED			DASH		
	Q1^a^	Q3	Q5	Q1	Q3	Q5	Q1	Q3	Q5
Mother–child pairs (n)	538	565	548	542	526	671	504	639	492
Ethnicity (%)									
White	98.7	98.1	98.2	98.7	98.9	98.5	97.4	98.6	99.0
Maternal total energy intake, mean (SD)	2095 (513)	1952 (536)	1891 (556)	1672 (498)	1939 (497)	2263 (519)	1678 (522)	1989 (531)	2225 (485)
Maternal age at delivery, mean (SD)	31.7 (3.3)	33.0 (3.4)	33.9 (3.7)	31.9 (3.6)	32.7 (3.6)	33.6 (3.6)	32.2 (3.7)	32.8 (3.4)	33.7 (3.5)
BMI before pregnancy,^c^ mean (SD)	23.0 (4.2)	22.7 (3.7)	22.4 (3.0)	23.1 (4.3)	23.1 (3.9)	22.2 (3.2)	23.0 (4.3)	22.9 (4.1)	22.1 (3.2)
Non-drinkers (%)^c,d^	68.3	45.6	28.2	56.0	54.4	37.0	51.0	48.3	42.4
Physical activity^b^, min/week (%)^c^									
0	27.5	17.3	8.8	26.9	15.2	11.4	31.3	15.1	9.4
1–149	50.4	40.3	35.3	43.0	43.5	38.4	43.5	44.9	32.7
150–299	13.0	21.2	23.8	15.3	21.5	22.4	13.3	20.6	23.3
≥ 300	9.1	21.2	32.0	14.7	19.8	27.8	12.0	19.4	34.5
Smoking history before pregnancy^b^ (%)^c^									
Never	81.3	72.1	64.2	76.9	75,7	69.6	72.4	70.6	70.2
Past	11.5	21.1	30.6	15.0	17.4	26.0	16.0	23.7	25.2
Current	7.2	6.8	5.2	8.1	7.0	4.4	11.6	5.7	4.6
Husband’s education (%)^c^									
High school degree	42.6	29.4	20.4	38.0	34.3	23.3	43.2	31.3	22.4
College degree	34.1	36.0	33.7	34.5	37.0	35.1	33.1	34.9	32.2
Graduate school degree	23.3	34.7	45.9	27.5	28.7	41.6	23.7	33.8	45.4
Parity before first included pregnancy (%)^c^									
Nulliparous	21.1	23.4	21.0	22.6	23.8	19.4	17.9	21.3	23.0
One previous pregnancy	33.4	30.8	35.0	33.5	33.1	31.1	34.1	30.6	32.7
Two previous pregnancies	22.1	25.5	22.1	20.9	23.5	24.6	24.1	26.5	20.6
Three previous pregnancies	23.4	20.4	21.9	23.0	19.7	24.9	23.9	21.6	23.5
Gestational age (%)									
28–36 wks	7.5	7.8	7.3	7.5	6.7	6.9	10.1	5.6	6.0
37–41 wks	87.9	87.7	88.1	89.1	89.2	88.4	85.4	87.7	89.2
42 + wks	4.7	4.5	4.6	3.4	4.1	4.7	4.6	6.7	4.9
Offspring gender (%)									
Male	41.8	44.8	44.2	46.1	45.8	46.4	44.8	46.0	46.5
Female	58.2	55.2	55.8	53.9	54.2	53.6	55.2	54.0	53.5
Offspring age at GUTSII baseline 2004, mean (SD)	13.2 (0.5)	13.2 (0.5)	13.2 (0.6)	13.2 (0.6)	13.2 (0.5)	13.2 (0.5)	13.2 (0.6)	13.2 (0.5)	13.2 (0.5)

AHEI, Alternative Healthy Eating Index; aMed alternate Mediterranean; DASH, Dietary approaches to stop hypertension; SD, standard deviation

^a^For simplicity, only quintiles 1, 3 and 5 are shown, Q1 refers to the lowest and Q5 to the highest diet quality. ^b^Recorded on the most recent questionnaire prior to conception of first included offspring. ^c^Percentages are of non-missing values. ^d^Recorded as part of food frequency questionnaire in 1991

**Table 2:** Relative risks (RR) and 95% confidence intervals (95% CI) for offspring ever having overweight or obesity during follow-up across quintiles of maternal dietary patterns during peripregnancy using data from 2702 participants of the Growing Up Today Study 2 from 2004 to 2013, restricted to singleton births

	Q1	Q2	Q3	Q4	Q5	P trend
AHEI-2010						
Cases/participants	184/530	190/550	199/560	160/520	180/542	
Basic model^a^	1.00 (ref.)	0.97 (0.82; 1.14)	1.01 (0.85; 1.18)	0.84 (0.71; 1.01)	0.93 (0.79; 1.10)	0.19
MV Model 1^b^	1.00 (ref.)	0.96 (0.82; 1.12)	1.03 (0.88; 1.21)	0.85 (0.72; 1.01)	0.95 (0.81; 1.13)	0.34
MV Model 2^c^	1.00 (ref.)	0.96 (0.82; 1.13)	1.08 (0.92; 1.26)	0.89 (0.75; 1.06)	1.03 (0.86; 1.22)	0.94
aMED						
Cases/participants	197/533	166/488	190/519	158/495	202/667	
Basic model^a^	1.00 (ref.)	0.91 (0.77; 1.08)	0.99 (0.85; 1.16)	0.87 (0.73; 1.04)	0.82 (0.70; 0.97)	0.02
MV Model 1^b^	1.00 (ref.)	0.92 (0.78; 1.08)	1.01 (0.86; 1.18)	0.88 (0.74; 1.04)	0.88 (0.75; 1.05)	0.14
MV Model 2^c^	1.00 (ref.)	0.92 (0.79; 1.09)	1.03 (0.88; 1.20)	0.90 (0.76; 1.08)	0.93 (0.78; 1.10)	0.40
DASH						
Cases/participants	172/500	197/519	217/630	182/566	145/487	
Basic model^a^	1.00 (ref.)	1.09 (0.92; 1.28)	0.99 (0.84; 1.17)	0.93 (0.78; 1.11)	0.86 (0.72; 1.04)	0.04
MV Model 1^b^	1.00 (ref.)	1.09 (0.93; 1.28)	1.00 (0.85; 1.18)	0.98 (0.82; 1.17)	0.93 (0.77; 1.12)	0.27
MV Model 2^c^	1.00 (ref.)	1.11 (0.94; 1.30)	1.03 (0.88; 1.21)	1.03 (0.86; 1.23)	0.98 (0.81; 1.19)	0.69

AHEI, Alternative Healthy Eating Index; aMed alternate Mediterranean; DASH, Dietary approaches to stop hypertension; CI, confidence interval; RR, relative risk; MV, multivariable model

^a^Adjusted for maternal total energy intake (continuous), offspring sex (boy/girl) and gestational age (28–36, 37–41, ≥ 42 wks). ^b^Additionally adjusted for BMI before pregnancy (< 18.5, 18, 0.5 < 25, 25–29, ≥ 30 kg/m^2^), smoking status before pregnancy (never, current, past), physical activity (0, 1–149, 150–299, ≥ 300 min/week of moderate to vigorous intensity). ^c^Additionally adjusted for maternal age at pregnancy, parity (nulliparous, 1, 2, 3 + previous pregnancies), husband’s education (less than 2 yr college, 4 yr college, graduate school)

**Table 3:** Relative risks (RR) and 95% confidence intervals (95% CI) for offspring ever having overweight or obesity during follow-up across quintiles of maternal dietary patterns during peripregnancy using data from 2282 participants of the Growing Up Today Study 2 from 2004 to 2013, restricted to singleton births, startified by maternal BMI before pregnancy

	Q1	Q2	Q3	Q4	Q5	P trend
AHEI-2010						
Mothers having normal weight (BMI < 25 kg/m^2^)						
Cases/participants	97/360	105/366	123/397	94/346	108/378	
Basic model^a^	1.00 (ref.)	1.02 (0.81; 1.29)	1.12 (0.90; 1.40)	0.94 (0.73; 1.20)	1.04 (0.83; 1.32)	0.97
MV Model 2^b^	1.00 (ref.)	1.05 (0.84; 1.33)	1.19 (0.95; 1.50)	1.02 (0.79; 1.31)	1.16 (0.91; 1.49)	0.32
MV Model 3^c^	1.00 (ref.)	1.09 (0.87; 1.37)	1.19 (0.95; 1.49)	1.04 (0.81; 1.33)	1.16 (0.91; 1.48)	0.34
Mothers having overweight or obesity (BMI ≥ 25 kg/m^2^)						
Cases/participants	55/93	55/101	49/81	40/80	38/80	
Basic model^a^	1.00 (ref.)	0.92 (0.71; 1.16)	1.02 (0.80; 1.30)	0.86 (0.65; 1.14)	0.78 (0.59; 1.04)	0.09
MV Model 2^b^	1.00 (ref.)	0.89 (0.70; 1.12)	1.05 (0.82; 1.35)	0.89 (0.68; 1.18)	0.81 (0.61; 1.09)	0.22
MV Model 3^c^	1.00 (ref.)	0.89 (0.70; 1.23)	1.05 (0.82; 1.35)	0.90 (0.68; 1.19)	0.82 (0.61; 1.11)	0.26
	P interaction^d^ = 0.51					
aMED						
Mothers having normal weight (BMI < 25 kg/m^2^)						
Cases/participants	112/365	91/329	111/351	83/327	130/475	
Basic model^a^	1.00 (ref.)	0.88 (0.70; 1.11)	1.03 (0.83; 1.29)	0.84 (0.66; 1.07)	0.90 (0.72; 1.13)	0.34
MV Model 2^b^	1.00 (ref.)	0.88 (0.70; 1.11)	1.06 (0.85; 1.33)	0.87 (0.68; 1.12)	0.97 (0.68; 1.22)	0.81
MV Model 3^c^	1.00 (ref.)	0.89 (0.70; 1.12)	1.04 (0.84; 1.29)	0.91 (0.71; 1.17)	1.02 (0.81; 1.29)	0.75
Mothers having overweight or obesity (BMI ≥ 25 kg/m^2^)						
Cases/participants	54/101	47/75	48/85	50/91	38/83	
Basic model^a^	1.00 (ref.)	1.16 (0.90; 1.50)	1.04 (0.80; 1.36)	1.02 (0.78; 1.33)	0.85 (0.61; 1.16)	0.26
MV Model 2^b^	1.00 (ref.)	1.16 (0.89; 1.50)	1.06 (0.80; 1.39)	1.05 (0.80; 1.38)	0.92 (0.67; 1.28)	0.54
MV Model 3^c^	1.00 (ref.)	1.16 (0.89; 1.50)	1.06 (0.80; 1.39)	1.05 (0.80; 1.39)	0.93 (0.67; 1.30)	0.60
	P interaction^d^ = 0.36					
aMED						
Mothers having normal weight (BMI < 25 kg/m^2^)						
Cases/participants	93/340	114/346	120/421	109/399	91/341	
Basic model^a^	1.00 (ref.)	1.19 (0.95; 1.50)	1.03 (0.82; 1.20)	1.00 (0.79; 1.27)	0.99 (0.76; 1.27)	0.52
MV Model 2^b^	1.00 (ref.)	1.22 (0.97; 1.54)	1.13 (0.89; 1.43)	1.12 (0.87; 1.44)	1.10 (0.84; 1.44)	0.67
MV Model 3^c^	1.00 (ref.)	1.25 (1.00; 1.57)	1.16 (0.92; 1.47)	1.12 (0.88; 1.44)	1.18 (0.90; 1.54)	0.42
Mothers having overweight or obesity (BMI ≥ 25 kg/m^2^)						
Cases/participants	46/81	50/89	67/113	45/87	29/65	
Basic model^a^	1.00 (ref.)	1.01 (0.77; 1.31)	1.03 (0.81; 1.32)	0.92 (0.69; 1.23)	0.76 (0.54; 1.06)	0.12
MV Model 2^b^	1.00 (ref.)	1.07 (0.82; 1.40)	1.08 (0.84; 1.40)	1.02 (0.75; 1.39)	0.86 (0.60; 1.23)	0.48
MV Model 3^c^	1.00 (ref.)	1.08 (0.81; 1.41)	1.09 (0.84; 1.40)	1.02 (0.75; 1.40)	0.87 (0.61; 1.25)	0.53
	P interaction^d^ = 0.79					

AHEI, alternative healthy eating index; aMed alternate Mediterranean; DASH, dietary approaches to stop hypertension; CI, confidence interval; RR, relative risk; MV, multivariable model

^a^Adjusted for maternal total energy intake (continuous), offspring sex (boy/girl) and gestational age (28–36, 37–41, ≥ 42 wks). ^b^Additionally adjusted for smoking status before pregnancy (never, current, past), physical activity (0, 1–149, 150–299, ≥ 300 min/week of moderate to vigorous intensity), maternal age at pregnancy, parity (nulliparous, 1, 2, 3 + previous pregnancies), husband’s education (less than 2 yr college, 4 yr college, graduate school). ^c^Additionally adjusted for continuous BMI (kg/m^2^). ^d^Obtained from MV Model 2

### Final sample for analyses

Since maternal diet was assessed for the first time in 1991 while the 10,918 children enrolled in GUTSII were born between 1987 and 1995, we only considered children born surrounding the 1991 diet assessment in our analyses. More specifically, we estimated the time of conception based on the children´s date of birth and based on that selected mother–child pairs where—based on the questionnaire return date—at least part of the pregnancy was covered by the 12 month period that the FFQ was enquiring about (n = 3085 children, n = 3008 mothers). We further restricted the sample to singleton births (n = 2991 children, n = 2954 mothers), children born after at least 28 weeks of gestation (n = 2768 children, n = 2737 mothers) and children with complete maternal exposure information (n = 2760 children, n = 2729 mothers). If two pregnancies of the same mother were covered partially by the 12 months period, we included only the first pregnancy which left us with a total sample of n = 2729 children, born to n = 2729 mothers for analyses. The sample size varies slightly by analysis based on availability of the considered outcomes. The children were aged 12–14 years at baseline in 2004 and 21–23 years at the last follow-up in 2013.

### Statistical analysis

Baseline descriptive characteristics were derived for all mother–child pairs eligible for the considered analyses across quintiles of the 3 dietary pattern scores. We estimated mean differences (MD) and 95% confidence intervals (CIs) in offspring birth weight across quintiles of dietary pattern scores considering the lowest quintile as the reference category. We further calculated relative risks (RR) and 95% CIs of offspring reporting a larger than median somatotype at age 5, offspring ever having overweight or obesity, and offspring ever having obesity. Due to the frequency of the considered outcomes we used log-binomial models to estimate RRs and approximations based on Poisson models with robust variance estimators in case of convergence problems [42].

Basic models were adjusted for total energy intake of the mothers, gestational age at delivery, offspring baseline age and sex. We further considered different sets of additional covariates. First, we added maternal pre-pregnancy lifestyle factors to the model, including pre-pregnancy BMI, smoking status and physical activity [multivariable (MV) model 1]. Second, we added maternal age at delivery, number of previous pregnancies and husband’s education (MV model 2). Since maternal pre-pregnancy BMI was previously reported a strong predictor of adverse offspring weight outcomes during adolescence [12], we also investigated potential effect modification by BMI. P values for interactions were based on score tests for testing multiplicative interactions of categorical variables. We introduced missingness indicators for covariates with missing values. All statistical tests were two sided and were considered statistically significant at *p* < 0.05. All analyses were conducted using SAS version 9.4 (SAS Institute, Cary, NC).

## Results

The average maternal age at birth was 32.8 (SD = 3.6) years (Table 1). Mothers with higher adherence to any of the healthful dietary patterns had a lower BMI, were more physically active and were less likely to be current smokers. In addition, women in the highest quintiles of adherence were more likely to be married to husbands with a graduate degree. Their offspring were on average 13.2 (SD = 0.6) years old at enrollment into the GUTSII study. More female offspring (53.6%) than males (46.4%) were included in the analysis.

In basic models adjusted for maternal total energy intake, offspring sex and gestational age, offspring of mothers with higher adherences to the aMED and DASH patterns were less likely to develop overweight or obesity during follow-up (P for trend: 0.02 for aMED and 0.04 for DASH) (Table 2). Compared to offspring born to mothers with the lowest quintile of aMED and DASH, the relative risks for having overweight or obesity were RR_Q5 vs Q1_ = 0.82 [0.70–0.97] and 0.86 [0.72–1.04] among offspring born to mothers with the highest quintiles of these dietary patterns scores. However, after adjustment for additional confounders, including maternal lifestyle factors, pre-pregnancy BMI and socioeconomic status, the effect estimates were attenuated and were no longer significant for any of the three dietary patterns (RR_Q5 vs Q1_ = 1.03 [0.86; 1.22] for AHEI, 0.93 [0.78; 1.10] for aMED and 0.98 [0.81; 1.19] for DASH, all *p*-trend > 0.05; Table 2). There were no associations between maternal adherence to the three healthful dietary patterns and offspring risk for obesity (Supplemental Table 1; RR_Q5 vs Q1_ = 0.92 [0.63; 1.35] for AHEI, 1.06 [0.72; 1.57] for aMED and 1.00 [0.65; 1.54] for DASH, all *p*-trend > 0.05). Thus, after adjustment for pre-pregnancy BMI and lifestyle factors, we did not observe any associations between maternal adherence to healthful dietary patterns and offspring weight outcomes during childhood until early adulthood.

Birth weight appeared to be similarly distributed across quintiles of maternal AHEI, aMED and DASH (Supplemental Table 2). Likewise, the risk of having a larger than median somatotype at age 5 was similar across quintiles of maternal dietary pattern scores (Supplemental Table 3).

We then investigated whether associations between maternal dietary patterns and offspring weight outcomes differed between mothers having normal-weight and those having overweight or obesity. Formal tests for multiplicative interactions showed no indication for effect modification (p_interaction_ for all outcomes > 0.05). Relative risks for offspring overweight or obesity stratified by maternal weight status are shown in Table 3.

In Supplemental Table 4 we present results for the association between adherences to dietary patterns and offspring overweight or obesity among mothers who did not drink any alcohol. The observed results were similar to those reported in Table 2.

## Discussion

In this cohort study of generally well-nourished mothers and their children, we found that adherence to healthy dietary patterns during the period surrounding pregnancy was not related to offspring weight outcomes from birth through early adulthood. While in age-adjusted analysis, greater maternal adherence to aMED and DASH (but not AHEI) tended to be associated with a lower risk of overweight or obesity in the offspring, none of the three dietary pattern scores remained significantly associated with offspring overweight or obesity risk after further adjustment for maternal pre-pregnancy BMI and lifestyle factors before pregnancy.

There are few published data on the associations between maternal healthful dietary patterns during pregnancy and weight outcomes in children of any age, yet our results align with those few that are available. Among 2695 Dutch mother–child pairs, significant inverse associations were observed between maternal adherences to 2 principal component derived dietary patterns that mainly consisted of healthy components and offspring BMI, fat mass index, and the risk of childhood overweight at age 6 years; however none of these associations remained significant after adjustment for socio-demographic and lifestyle factors of the mothers and children [30]. In the Dutch cohort, no independent associations were found between maternal dietary patterns during pregnancy and offspring blood lipids, blood pressure or insulin levels [43]. Among 1827 mother–child pairs from the Spanish ‘Infancia y Medio Ambiente’ cohort study, higher adherence to the Mediterranean diet in pregnancy was not associated with offspring overweight at age 4 years, but there was some evidence of an inverse association between diet quality in pregnancy and offspring waist circumference, a marker of abdominal obesity [29]. To summarize, results from our study and from the aforementioned studies suggest that maternal healthful dietary patterns during pregnancy are not consistently associated with offspring overweight or obesity risk during childhood, adolescence or young adulthood.

These findings do not imply that maternal diet quality during pregnancy would be of negligible importance; they only suggest that these maternal healthful dietary patterns are not directly associated with offspring weight outcomes after adjustment for maternal BMI and other lifestyle behaviors. However, in adult women, healthful dietary patterns are associated with less BMI gain over time [26], especially in genetically susceptible individuals [44]. Thus, it is likely that healthful maternal dietary patterns exert positive effects on offspring weight outcomes through a healthier maternal weight status before pregnancy. Indeed, in the present analysis, the inverse association between the aMED and DASH with offspring overweight risk was no longer observed after adjusting for maternal BMI and lifestyle factors before pregnancy. In addition, a previous study has shown that adherence to healthful dietary patterns as part of an overall healthy lifestyle before pregnancy is associated with a lower risk of gestational diabetes [45] and offspring obesity risk [14].

In the present study, we have focused on these well-validated healthful dietary patterns, but results might differ for maternal dietary patterns that are particularly “obesogenic” or for Western dietary patterns that are especially high in saturated fat or added sugars. For example, among the single nutrients that are currently suspected to be involved in prenatal programming of obesity are prenatal sugar and saturated fat intake [18], dietary glycemic index and glycemic load [19], dihomo-gamma-linolenic acid [46] and protein intake from animal sources [20]. Furthermore, associations between higher maternal intakes of sugar-sweetened beverages [21, 47] and higher offspring BMI have also been reported. Murrin et al. [18] showed that a dietary pattern scoring higher in processed foods during pregnancy, comprising of soft drinks, chips, roast potatoes, crisps, pizza, processed meat, sweets and chocolate, was significantly associated with offspring overweight and obesity at the age of 5 years.

We also examined birth weight as an outcome and did not find any associations between maternal diet quality score during the period surrounding pregnancy and offspring birth weight. In line with the published literature, studies on healthy dietary patterns, including the HEI and Mediterranean diet, generally report a lack of an association between maternal diet quality indices and offspring birth weight [28, 48, 49]. However, Rodríguez-Bernal et al. [28] observed an association between the HEI-2010 with higher fat mass but not fat-free mass or overall birth weight, thus an association with neonatal adiposity. The Growing Up in Singapore Towards healthy Outcomes cohort study came to the same conclusion; a higher maternal diet quality during pregnancy as measured by the HEI was associated with longer birth length and lower neonatal adiposity but not with birth weight and preterm birth [49].

Our study has several strengths, including the rather large sample size, the longitudinal follow-up of both mothers and their children, the availability of maternal dietary assessments via FFQ, and well phenotyped maternal profiles in terms of their lifestyle and other risk factors before and surrounding the time of pregnancy. No study to date has examined maternal dietary patterns in relation to offspring weight beyond childhood; the long follow-up of the children covering the ages between 12 and 23 years is therefore novel. The use of the diet quality indices can be seen as an advantage because they capture overall diet quality taking into account the complex nature of the habitual diet [23]; however, any potential effects of individual dietary components [50] could be cancelled out by opposing effects of individual nutrients or food groups.

There are some important study limitations to note. The FFQ was not modified for use during pregnancy and it was not necessarily administered during pregnancy; we only selected mother–child pairs where at least a part of the pregnancy was covered by the 12 months period the FFQ was enquiring about. Although it has been suggested that dietary patterns change little from before to during pregnancy [51], we cannot exclude the possibility that maternal dietary patterns have changed during pregnancy in our population. Our study population was comprised of predominantly white US nurses of a slightly higher socioeconomic status than the general population. Mothers in our study sample were in their early thirties when they gave birth, which is slightly above the national average birth age. Thus, our results might not be generalizable to other ethnic or socioeconomic groups, younger mothers or populations where malnutrition is still common.

Further, while we had detailed information on maternal factors that could potentially confound our results, we had no information on paternal factors, with the exception of their education—though husband’s education is considered one of the strongest proxies for socioeconomic status of the family. The influence of paternal dietary patterns in relation to pregnancy and offspring outcomes represents a novel topic warranting future investigation [52]. All weight measures were self-reported in our study. Comparisons of measured versus self-reported weight and height in US adolescents suggest a slight tendency for underreporting among adolescents with obesity [53–55]. Such misclassification, if it does exist, might have contributed to an attenuation of the associations examined. We also acknowledge the lack of more detailed adiposity measures as another limitation of this study.

Finally, moderate alcohol intake is considered healthy as a part of an overall healthy dietary pattern and therefore scored positively in the aMED and aHEI [36]. This scoring is contradictory to current dietary guidelines that recommend pregnant women to completely abstain from alcohol [56, 57]. The associations for alcohol and nutritional behaviors are difficult to disentangle, because moderate alcohol drinkers have healthier diets compared to heavy drinkers and abstainers, while heavy drinkers are more often characterized by higher intakes of high-fat meat and lower intake of cereals and fruit [58]. This clustering of health behaviors has been shown to persist into pregnancy, i.e. light-to-moderate alcohol consumption is associated with adherence to health conscious dietary patterns, while higher alcohol intake and binge drinking is related to higher intake of processed foods during pregnancy [59]. It is an advantage of the dietary pattern analysis to parallel more closely the real world, and take into account complex combinations of nutrients and possible joint and interactive effects [23]. Nonetheless, we conducted a sensitivity analysis among non-drinkers, and associations between adherence to healthful dietary patterns during peripregnancy and offspring risk of overweight were unchanged.

In conclusion, in this well characterized mother–child cohort, we did not find any evidence for an impact of healthful maternal dietary patterns during the period surrounding pregnancy on weight outcomes in their offspring during childhood through early adulthood. Maternal BMI and maternal lifestyle factors prior to pregnancy appeared to be confounders in these associations. Further studies on maternal nutrition during pregnancy and offspring health are warranted in different populations so that women of reproductive age can be provided with evidence-based dietary recommendations that may help to reduce the risk of overweight in their children during later life.

## Electronic supplementary material

Below is the link to the electronic supplementary material.

Supplementary material 1 (DOCX 25 kb)

## Abbreviations

AHEI: Alternate Healthy Eating Index
aMED: Alternate Mediterranean Diet
BMI: Body mass index
CIs: 95% confidence intervals
DASH: Dietary Approach to Stop Hypertension
FFQ: Food frequency questionnaire
GUTSII: Growing Up Today Study II
MD: Mean differences
MV: Multivariable
NHSII: Nurses’ Health Study II
RRs: Relative risks
US: United States
