# Supplementary material for: Maternal healthful dietary patterns during peripregnancy and long-term overweight risk in their offspring
Source: Eur J Epidemiol. 2020 Mar 17;35(3):283–93. doi: 10.1007/s10654-020-00621-8 (PMC7154013; doi:10.1007/s10654-020-00621-8)
Supplement: Supplementary file 1 — Supplementary material 1 (DOCX 25 kb) [file 10654_2020_621_MOESM1_ESM.docx]

**Supplemental Table 1: Relative risks (RR) for offspring ever having obesity during follow-up across quintiles of maternal dietary patterns during peripregnancy using data from 2,702 participants of the Growing Up Today Study 2 from 2004 to 2013, restricted to singleton births.**

|  | Q1 | Q2 | Q3 | Q4 | Q5 | P trend |
| --- | --- | --- | --- | --- | --- | --- |
| **AHEI-2010** |  |  |  |  |  |  |
| Cases/participants | 54/530 | 58/550 | 56/560 | 46/520 | 45/542 |  |
| Basic model ^a^ | 1.00 (ref.) | 0.99 (0.70; 1.41) | 0.95 (0.66; 1.35) | 0.80 (0.55; 1.18) | 0.77 (0.53; 1.13) | 0.09 |
| MV Model 1 ^b^ | 1.00 (ref.) | 0.95 (0.68; 1.34) | 1.00 (0.71; 1.42) | 0.82 (0.57; 1.19) | 0.84 (0.58; 1.23) | 0.26 |
| MV Model 2 ^c^ | 1.00 (ref.) | 0.95 (0.68; 1.33) | 1.06 (0.75; 1.50) | 0.86 (0.60; 1.24) | 0.92 (0.63; 1.35) | 0.55 |
| **aMED** |  |  |  |  |  |  |
| Cases/participants | 55/533 | 52/488 | 48/519 | 52/495 | 52/667 |  |
| Basic model ^a^ | 1.00 (ref.) | 1.03 (0.72; 1.48) | 0.93 (0.64;1.35) | 1.08 (0.75; 1.55) | 0.82 (0.56; 1.21) | 0.40 |
| MV Model 1 ^b^ | 1.00 (ref.) | 1.09 (0.77; 1.55) | 0.98 (0.68; 1.40) | 1.13 (0.79; 1.62) | 1.00 (0.68; 1.46) | 0.93 |
| MV Model 2 ^c^ | 1.00 (ref.) | 1.11 (0.78; 1.57) | 0.99 (0.69; 1.43) | 1.19 (0.83; 1.69) | 1.06 (0.72; 1.57) | 0.66 |
| **DASH** |  |  |  |  |  |  |
| Cases/participants | 53/500 | 52/519 | 56/630 | 61/566 | 37/487 |  |
| Basic model ^a^ | 1.00 (ref.) | 0.95 (0.66; 1.36) | 0.86 (0.60; 1.23) | 1.08 (0.75; 1.55) | 0.77 (0.51; 1.17) | 0.49 |
| MV Model 1 ^b^ | 1.00 (ref.) | 0.96 (0.68; 1.37) | 0.87 (0.61; 1.24) | 1.18 (0.83; 1.69) | 0.90 (0.59; 1.37) | 0.91 |
| MV Model 2 ^c^ | 1.00 (ref.) | 1.00 (0.70; 1.42) | 0.92 (0.64; 1.30) | 1.30 (0.90; 1.88) | 1.00 (0.65; 1.54) | 0.51 |

Abbreviations: AHEI, Alternative Healthy Eating Index; aMed alternate Mediterranean; DASH, Dietary approaches to stop hypertension; CI, confidence interval; RR, relative risk; MV, multivariable model

^a^ Adjusted for maternal total energy intake (continuous), offspring sex (boy/girl) and gestational age (28 - 36, 37 - 41, ≥ 42 wks)

**^b^** Additionally adjusted for BMI before pregnancy (< 18.5, 18,.5 < 25, 25-29, ≥ 30 kg/m^2^), smoking status before pregnancy (never, current, past), physical activity (0, 1-149, 150 -299, ≥ 300 min/week of moderate to vigorous intensity)

^c^ Additionally adjusted for maternal age at pregnancy, parity (nulliparous, 1, 2, 3+ previous pregnancies), husband’s education (less than 2yr college, 4yr college, graduate school)

**Supplemental Table 2. Adjusted mean differences (MD) in offspring birth weight (gram) across quintiles of maternal dietary patterns during peripregnancy using data from 2,223 participants of the Growing Up Today Study 2 from 2004 to 2013, restricted to singleton birth.**

|  | Q1 | Q2 | Q3 | Q4 | Q5 | P trend |
| --- | --- | --- | --- | --- | --- | --- |
| **AHEI-2010** | MD (95 % CI) | | | | |  |
| Participants | 452 | 446 | 464 | 427 | 434 |  |
| Basic model ^a^ | 0 (ref) | -64.13 (-131.00; 2.73) | -51.06 (-117.23; 15.11) | -49.17 (-117.20; 18.85) | -70.67 (-138.13; -3.21) | 0.09 |
| MV Model 1 ^b^ | 0 (ref) | -62.36 (-129.02; 4.30) | -44.94 (-111.11; 21.22) | -48.70 (-116.87; 19.47) | -63.79 (-131.86; 4.28) | 0.13 |
| MV Model 2 ^c^ | 0 (ref) | -67.27 (-134.38; -0.16) | -50.80 (-118.58; 16.98) | -58.24 (-128.99; 12.51) | -69.8 (-141.73; 2.78) | 0.11 |
| **aMED** | MD (95 % CI) | | | | |  |
| Participants | 456 | 387 | 426 | 411 | 543 |  |
| Basic model ^a^ | 0 (ref) | -18.27 (-87.81; 51.27) | -17.44 (-85.72; 50.84) | -23.84 (-94.02; 46.33) | -21.50 (-89.99; 46.98) | 0.55 |
| MV Model 1 ^b^ | 0 (ref) | -14.48 (-83.91; 54.94) | -18.32 (-86.48; 49.85) | -20.82 (-91.16; 49.52) | -14.45 (-83.40; 54.50) | 0.67 |
| MV Model 2 ^c^ | 0 (ref) | -18.54 (-88.11; 51.03) | -23.61 (-92.64; 45.41) | -29.66 (-101.46; 42.15) | -22.16 (-93.55; 49.23) | 0.53 |
| **DASH** | MD (95 % CI) | | | | |  |
| Participants | 415 | 425 | 536 | 448 | 399 |  |
| Basic model ^a^ | 0 (ref) | 5.15 (-64.31; 74.60) | -1.56 (-68.50; 65.38) | 43.02 (-27.75; 113.79) | 51.63 (-22.30; 125.56) | 0.13 |
| MV Model 1 ^b^ | 0 (ref) | 1.77 (-67.51; 71.05) | -6.09 (-73.08; 60.90) | 34.99 (-35.99; 105.8) | 52.63 (-21.53; 126.80) | 0.17 |
| MV Model 2 ^c^ | 0 (ref) | 4.34 (-65.38; 74.06) | -5.55 (-73.77; 62.67) | 39.12 (-34.11; 112.35) | 63.10 (-14.35; 140.55) | 0.11 |

Abbreviations: AHEI, Alternative Healthy Eating Index; aMed alternate Mediterranean; DASH, Dietary approaches to stop hypertension; CI, confidence interval; MD, mean difference; MV, multivariable model

^a^ Adjusted for maternal total energy intake (continuous), offspring sex (boy/girl) and gestational age (28 - 36, 37 - 41, ≥ 42 wks)

**^b^** Additionally adjusted for BMI before pregnancy (< 18.5, 18,.5 < 25, 25-29, ≥ 30 kg/m^2^), smoking status before pregnancy (never, current, past), physical activity (0, 1-149, 150 -299, ≥ 300 min/week of moderate to vigorous intensity)

^c^ Additionally adjusted for maternal age at pregnancy, parity (nulliparous, 1, 2, 3+ previous pregnancies), husband’s education (less than 2yr college, 4yr college, graduate school)

**Supplemental Table 3: Relative risks (RR) for larger than median somatotype at age 5 across quintiles of maternal dietary patterns during peripregnancy using data from 2,635 participants of the Growing Up Today Study 2 from 2004 to 2013, restricted to singleton births.**

|  | Q1 | Q2 | Q3 | Q4 | Q5 | P trend |
| --- | --- | --- | --- | --- | --- | --- |
| **AHEI-2010** | RR (95 % CI) | | | | |  |
| Cases/participants | 194/525 | 225/536 | 223/545 | 210/508 | 217/521 |  |
| Basic model ^a^ | 1.00 (ref.) | 1.12 (0.96; 1.30) | 1.10 (0.95; 1.28) | 1.10 (0.94; 1.28) | 1.12 (0.97; 1.31) | 0.22 |
| MV Model 1 ^b^ | 1.00 (ref.) | 1.12 (0.96; 1.30) | 1.12 (0.96; 1.30) | 1.12 (0.96; 1.31) | 1.16 (0.99; 1.36) | 0.10 |
| MV Model 2 ^c^ | 1.00 (ref.) | 1.11 (0.96; 1.29) | 1.13 (0.97; 1.32) | 1.12 (0.96; 1.32) | 1.17 (1.00; 1.38) | 0.08 |
| **aMED** | RR (95 % CI) | | | | |  |
| Cases/participants | 219/524 | 183/479 | 209/506 | 200/476 | 258/650 |  |
| Basic model ^a^ | 1.00 (ref.) | 0.91 (0.78; 1.06) | 0.98 (0.85; 1.14) | 1.00 (0.86; 1.16) | 0.94 (0.84; 1.09) | 0.82 |
| MV Model 1 ^b^ | 1.00 (ref.) | 0.91 (0.78; 1.06) | 1.00 (0.86; 1.15) | 1.01 (0.87; 1.18) | 0.97 (0.84; 1.13) | 0.83 |
| MV Model 2 ^c^ | 1.00 (ref.) | 0.91 (0.78; 1.06) | 1.00 (0.86; 1.16) | 1.01 (0.87; 1.18) | 0.97 (0.83; 1.13) | 0.83 |
| **DASH** | RR (95 % CI) | | | | |  |
| Cases/participants | 189/486 | 220/512 | 249/615 | 218/546 | 193/476 |  |
| Basic model ^a^ | 1.00 (ref.) | 1.10 (0.94; 1.27) | 1.03 (0.89; 1.20) | 1.02 (0.87; 1.19) | 1.04 (0.88; 1.22) | 0.63 |
| MV Model 1 ^b^ | 1.00 (ref.) | 1.11 (0.95; 1.29) | 1.06 (0.91; 1.23) | 1.06 (0.90; 1.24) | 1.08 (0.92; 1.24) | 0.42 |
| MV Model 2 ^c^ | 1.00 (ref.) | 1.11 (0.95; 1.29) | 1.06 (0.91; 1.24) | 1.06 (0.90; 1.25) | 1.09 (0.92; 1.30) | 0.36 |

Abbreviations: AHEI, Alternative Healthy Eating Index; aMed alternate Mediterranean; DASH, Dietary approaches to stop hypertension; CI, confidence interval; RR, relative risk; MV, multivariable model

^a^ Adjusted for maternal total energy intake (continuous), offspring sex (boy/girl) and gestational age (28 - 36, 37 - 41, ≥ 42 wks)

**^b^** Additionally adjusted for BMI before pregnancy (< 18.5, 18,.5 < 25, 25-29, ≥ 30 kg/m^2^), smoking status before pregnancy (never, current, past), physical activity (0, 1-149, 150 -299, ≥ 300 min/week of moderate to vigorous intensity)

^c^ Additionally adjusted for maternal age at pregnancy, parity (nulliparous, 1, 2, 3+ previous pregnancies), husband’s education (less than 2yr college, 4yr college, graduate school)

**Supplemental Table 4: Relative risks (RR) for offspring ever having overweight or obesity during follow-up across quintiles of maternal dietary patterns during peripregnancy using data from the Growing Up Today Study 2 from 2004 to 2013, restricted to singleton births, among non-drinking mothers (n=1315).**

|  | Q1 | Q2 | Q3 | Q4 | Q5 | P trend |
| --- | --- | --- | --- | --- | --- | --- |
| **AHEI-2010** |  |  |  |  |  |  |
| Cases/participants | 134/363 | 115/322 | 100/256 | 81/222 | 46/152 |  |
| Basic model ^a^ | 1.00 (ref.) | 0.94 (0.78;1.16) | 1.04 (0.85; 1.28) | 0.94 (0.75; 1.18) | 0.79 (0.60; 1.05) | 0.18 |
| MV Model 1 ^b^ | 1.00 (ref.) | 0.96 (0.79; 1.16) | 1.07 (0.87; 1.31) | 0.97 (0.77; 1.20) | 0.84 (0.64; 1.10) | 0.37 |
| MV Model 2 ^c^ | 1.00 (ref.) | 0.97 (0.80; 1.17) | 1.12 (0.91; 1.37) | 1.00 (0.80; 1.25) | 0.90 (0.68; 1.18) | 0.75 |
| **aMED** |  |  |  |  |  |  |
| Cases/participants | 116/299 | 79/248 | 119/286 | 85/237 | 77/245 |  |
| Basic model ^a^ | 1.00 (ref.) | 0.82 (0.65; 1.03) | 1.09 (0.89; 1.33) | 0.96 (0.76; 1.20) | 0.84 (0.66; 1.07) | 0.47 |
| MV Model 1 ^b^ | 1.00 (ref.) | 0.83 (0.67; 1.05) | 1.20 (0.90; 1.34) | 0.95 (0.76; 1.19) | 0.92 (0.72; 1.18) | 0.88 |
| MV Model 2 ^c^ | 1.00 (ref.) | 0.85 (0.67; 1.06) | 1.10 (0.91; 1.34) | 0.99 (0.79; 1.24) | 0.97 (0.76; 1.25) | 0.72 |
| **DASH** |  |  |  |  |  |  |
| Cases/participants | 92/255 | 112/265 | 107/305 | 108/281 | 57/207 |  |
| Basic model ^a^ | 1.00 (ref.) | 1.17 (0.94; 1.45) | 0.97 (0.78; 1.22) | 1.07 (0.85; 1.34) | 0.77 (0.58; 1.03) | 0.11 |
| MV Model 1 ^b^ | 1.00 (ref.) | 1.15 (0.93; 1.42) | 0.95 (0.76; 1.18) | 1.10 (0.88; 1.38) | 0.82 (0.62; 1.09) | 0.27 |
| MV Model 2 ^c^ | 1.00 (ref.) | 1.16 (0.94; 1.43) | 0.97 (0.78; 1.21) | 1.15 (0.91; 1.44) | 0.87 (0.66; 1.16) | 0.56 |

Abbreviations: AHEI, alternative healthy eating index; aMed, alternate Mediterranean; DASH, Dietary approaches to stop hypertension; CI, confidence interval; RR, relative risk; MV, multivariable model

^a^ Adjusted for maternal total energy intake (continuous), offspring sex (boy/girl) and gestational age (28 - 36, 37 - 41, ≥ 42 wks)

**^b^** Additionally adjusted for BMI before pregnancy (< 18.5, 18,.5 < 25, 25-29, ≥ 30 kg/m^2^), smoking status before pregnancy (never, current, past), physical activity (0, 1-149, 150 -299, ≥ 300 min/week of moderate to vigorous intensity)

^c^ Additionally adjusted for maternal age at pregnancy, parity (nulliparous, 1, 2, 3+ previous pregnancies), husband’s education (less than 2yr college, 4yr college, graduate school)
